# Peripheral artery disease: a comparison of urgent open and endovascular revascularizations on the public health system in Brazil, from 2010 to 2020

**DOI:** 10.1590/1677-5449.202200161

**Published:** 2022-07-29

**Authors:** Thaís Rodrigues Magalhães, Daniel César Magalhães Fernandes, Roberto Gomide, Hideki Nakano, André Vespasiano Afiune, Rômulo Mendes Silva, Paulo Ricardo Alves Moreira, Rosa Tanmirys de Sousa Lima

**Affiliations:** 1 Hospital Geral de Goiânia Dr. Alberto Rassi – HGG, Goiânia, GO, Brasil.

**Keywords:** peripheral artery disease, open revascularization, endovascular surgery

## Abstract

**Background:**

Peripheral artery disease (PAD) has high prevalence and is associated with high risk of cardiovascular events. Surgical or endovascular intervention is necessary in chronic limb-threatening ischemia.

**Objectives:**

To evaluate the distribution of open and endovascular revascularizations in different regions of Brazil, analyzing the health system costs and mortality related to these procedures.

**Methods:**

A descriptive, cross-sectional, observational, epidemiological study was carried out to evaluate open and endovascular surgeries performed on the SUS public healthcare system in Brazil, from 2010 to 2020. Data were collected from the SUS Department of Informatics (Datasus).

**Results:**

Over the period analyzed, 83,218 admissions for open and endovascular surgeries were registered, with a total cost of R$ 333,989,523.17. There were more hospital admissions for percutaneous procedures (56,132) than for conventional surgery (27,086). Most of the procedures (83%) were performed in the country’s Southeast and South regions, while the North region had the lowest number of procedures. Over the period evaluated, there was a decreasing trend for open procedures and an increasing trend for endovascular procedures. The average hospital stay was shorter for endovascular procedures (5.3 days) than for open surgery (10.2 days). The analysis of mortality related to these procedures revealed a higher rate of in-hospital mortality associated with open revascularization than with endovascular (5.24% vs. 1.56%).

**Conclusions:**

Endovascular techniques constituted the primary approach for revascularization treatment in critical limb-threatening ischemia, with a lower in-hospital mortality rate and shorter hospital stay when compared to open surgeries.

## INTRODUCTION

Peripheral arterial occlusive disease (PAOD) is caused by systemic atherosclerotic phenomena that provoke arterial obstructions, is associated with high rates of cardiovascular morbidity and mortality, and affects a large proportion of the population, causing considerable human suffering and economic cost.^1^^,^^2^

From 2000 to 2010, the prevalence of PAOD increased by 13.1% in high-income countries and by 28.7% in medium and low income countries.^3^^,^^4^

Surgical treatment is indicated for patients with critical limb ischemia, whether manifest by pain at rest or trophic lesions, and for patients with intermittent claudication who do not respond to clinical treatment, with limitations affecting a quality of life and/or employment, assuming anatomic conditions permit the procedure.^1^

Patients who are candidates for surgical treatment can be treated with open or endovascular revascularization and, if it is impossible to salvage the limb, may need primary or secondary major amputations.^5^^-^8

Historically, revascularization was considered the gold standard for patients with occlusive peripheral arterial disease, with excellent limb salvage rates and durability.^9^

However, an endovascular revolution has occurred over the last two decades, with significant increases in use of percutaneous techniques for revascularization of ischemic limbs and countless reports of excellent limb salvage rates. Moreover, endovascular treatment has demonstrated lower perioperative morbidity and mortality than open techniques.^9^

It has therefore become important to determine the distribution of open and endovascular surgery, raising questions about the rates of admission, health system costs, and mortality related to these procedures. The objective of this study was thus to conduct a comparative analysis of surgical and endovascular revascularizations performed on the public healthcare system in Brazil’s five administrative regions from 2010 to 2020.

## METHOD

This is a descriptive, cross-sectional, observational, epidemiological study using data harvested from information on a Ministry of Health database maintained by Datasus (the IT department of the Sistema Única de Saúde [SUS]). Using the TABNET portal, the “Healthcare” section was selected, followed by the “Hospital Production (SIH/SUS)” subsection, and then the “Consolidated AIH (RD) data, by place of admission, from 2008” dataset, with the geographic coverage option “Brazil by Region and State”.

Data were extracted on admissions related to open and endovascular revascularizations from January 2010 to December 2020, selecting patients admitted on an urgent basis, in order to analyze procedures performed on patients with probable critical limb ischemia.

The codes selected for procedures related to open revascularization were: 0406020310; 0406020329; 0406020345; 0406020353; 0406020361; 0406020388; 0406020450; 0406020442; and 0406020434. The codes selected for endovascular procedures were: 0406040281; 0406040028; 0406040044; 0406040052; 0406040060; and 0406040079. Amputation codes were not selected because the open-access Datasus database does not enable differentiation between procedures performed because of trauma from those provoked by critical ischemia.

Data were also extracted on mean length of hospital stay, total expenditure per admission, and mortality rates related to the procedures conducted during the period. Admission rates were calculated by dividing the number of admissions by the population of residents per year and region and expressed as rates per 100,000 inhabitants, using data from the Brazilian Institute of Geography and Statistics (IBGE - Instituto Brasileiro de Geografia e Estatística). The results are presented as tables and graphs.

The Prais-Winsten method was used to conduct a temporal analysis of the results, employing a linear regression model executed in Stata to classify trends in the results as rising, falling, or static, by calculating p values (greater or less than 0.05) and b values (greater or less than 0).

Research Ethics Committee approval was waived because the data employed are in the public domain, with open and unrestricted access, and do not identify individual patients.

## RESULTS

In Brazil, data for the period from 2010 to 2020 show a total of 83,218 urgent admissions for open or endovascular revascularizations, with a total expenditure of R$ 333,989,523.17 on hospital stays and procedures. As shown in Table 1, the numbers of admissions and total hospital costs were highest in Brazil’s Southeast region and lowest in its North region.

**Table 1:** Number and total cost of urgent admissions for open and endovascular revascularizations by region of Brazil from 2010 to 2020.

**Region**	**Admissions**	**Total cost (Reais)**
North region	881	R$ 3,161,867.43
Northeast region	9,542	R$ 34,663,032.26
Southeast region	36,548	R$ 152,446,124.24
South region	32,314	R$ 127,179,973.15
Midwest region	3,933	R$ 16,538,526.09
**Total**	83,218	R$ 333,989,523.17

Source: Ministry of Health, Sistema Única de Saúde Hospital Information System (SIH/SUS).

**Figure 1:**
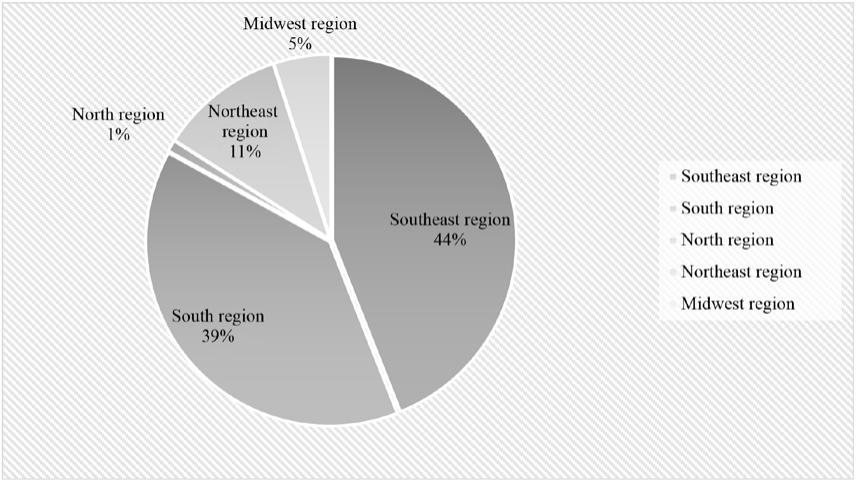
Relative frequency of total numbers of open and endovascular procedures by region of Brazil from 2010 to 2020 .

Figure 1 illustrates the approximate relative frequency of the total numbers of open and endovascular revascularizations conducted in each region of Brazil. It can be observed that the greater part (83%) of admissions were concentrated in the Southeast and South regions and only 1% of all procedures were conducted in the North region.

Table 2 shows that a total of 27,086 admissions were approved for open revascularization surgery from 2010 to 2020 in Brazil, at a total cost of R$ 94,614,711.94.

**Table 2:** Number of admissions, mean length of hospital stay, mortality rate, and total expenditure on urgent open revascularization by region of Brazil from 2010 to 2020.

**Region**	**Admissions**	**Mean stay (days)**	**Mortality rate (%)**	**Total cost** **(Reais)**
North region	228	13.3	7.89	R$ 674,170.87
Northeast region	1,755	13.1	5.87	R$ 6,355,481.17
Southeast region	13,420	9.5	5.98	R$ 49,605,675.58
South region	10,119	9.4	4.08	R$ 32,504,387.60
Midwest region	1,564	17.1	5.31	R$ 5,474,996.72
**Total**	27,086	10.2	5.24	R$ 94,614,711.94

Source: Ministry of Health, Sistema Única de Saúde Hospital Information System (SIH/SUS).

Once more, it was the Southeast and South regions that accounted for the largest proportion of the open revascularization, at a combined 87% of all such surgery conducted in Brazil. The North region was in last place, where only 228 open procedures were conducted during the period analyzed. Similarly, the total expenditure was in line with the numbers of admissions and procedures, highest in the Southeast region and lowest in the North region.

With relation to mean length of hospital stay for open revascularization, The Midwest region has the longest length of hospital stay (17.1 days) and the South region had the shortest mean stay (9.4 days). For Brazil as a whole, the mean hospital stay for open surgery was 10.2 days.

Analysis of the relative mortality of the open procedures revealed an overall in-hospital mortality rate of 5.24% for the whole of Brazil from 2010 to 2020. Despite having the lowest number of procedures, the North region had the highest mortality rate (7.89%), followed by the Southeast region (5.98%), Northeast region (5.87%), Midwest region (5.31%), and South region (4.08%).

**Figure 2:**
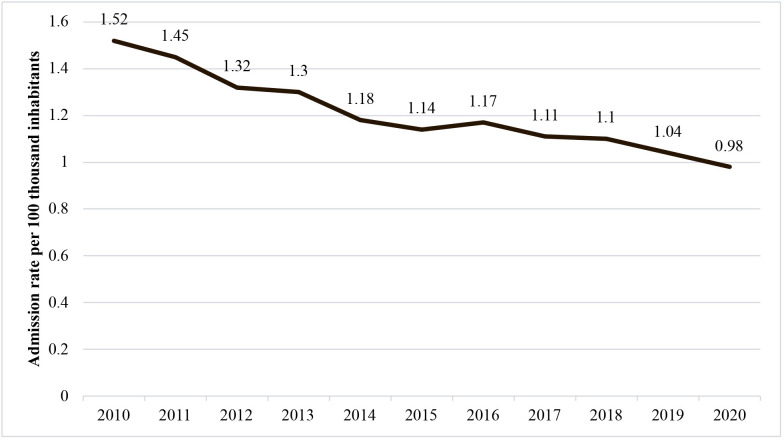
Rates of urgent admissions for open revascularization in Brazil from 2010 to 2020.

**Figure 3:**
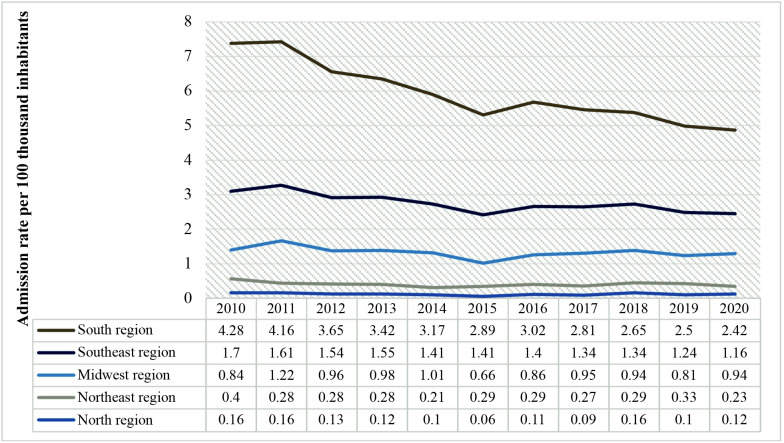
Distribution of rates of urgent admission for open revascularization by region of Brazil from 2010 to 2020.

Finally, calculating the rate of admissions for urgent open procedures, as illustrated in Figure 2, a non-static (p < 0.05) and falling (b < 0) trend was observed over the period analyzed for the whole of Brazil. Figure 3 illustrates urgent admission rates per year, by the country’s five regions.

Table 3 shows that there were 56,132 admissions for endovascular procedures in Brazil from 2010 to 2020, at a total cost of R$ 239,374,811.23.

**Table 3:** Number of admissions, mean length of hospital stay, mortality rate, and total expenditure on urgent endovascular surgery by region of Brazil from 2010 to 2020.

**Region**	**Admissions**	**Mean stay (days)**	**Mortality rate (%)**	**Total cost (Reais)**
North region	653	8.9	3.52	R$ 2,487,696.56
Northeast region	7,787	7.5	1.81	R$ 28,307,551.09
Southeast region	23,128	4.2	1.47	R$ 102,840,448.66
South region	22,195	5.1	1.41	R$ 94,675,585.55
Midwest region	2,369	9.1	2.57	R$ 11,063,529.37
**Total**	56,132	5.3	1.56	R$ 239,374,811.23

Source: Ministry of Health, Sistema Única de Saúde Hospital Information System (SIH/SUS).

The Southeast and South regions accounted for the great majority of these procedures in the country (80.7%), while just 1.2% of these angioplasties were conducted in the North region.

The mean length of hospital stay for endovascular procedures in Brazil was 5.3 days and stays were longest in the Midwest region (9.1 days) and shortest in the Southeast region (4.2 days).

**Figure 4:**
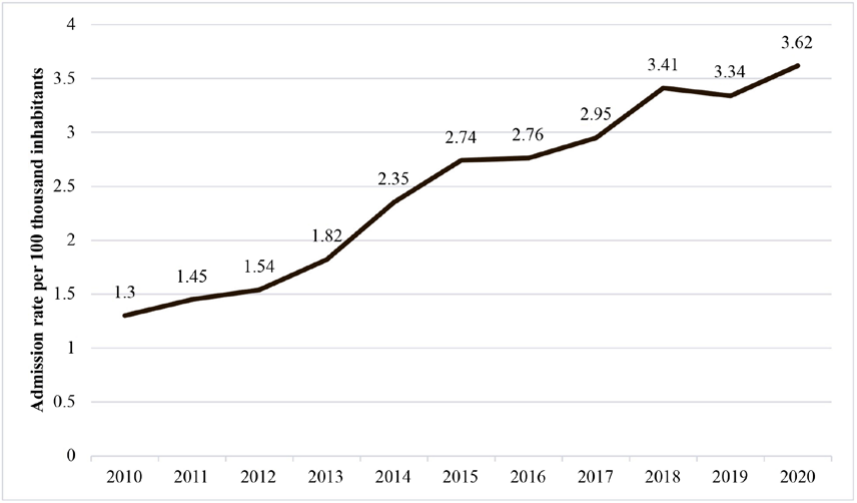
Rates of urgent admissions for endovascular revascularization in Brazil from 2010 to 2020.

**Figure 5:**
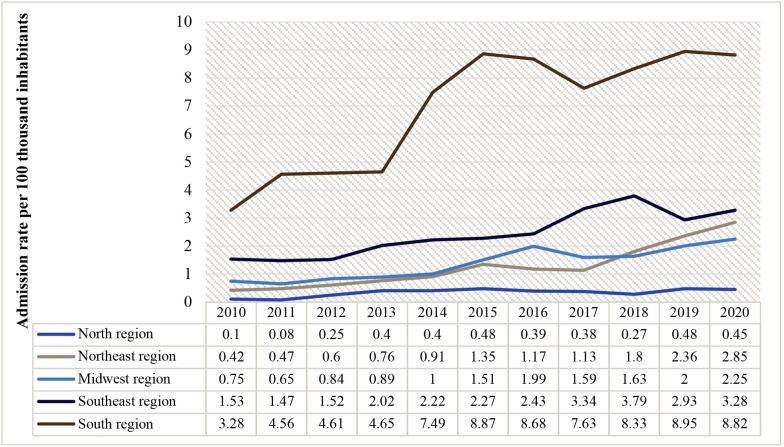
Distribution of rates of urgent admission for endovascular revascularization by region of Brazil from 2010 to 2020.

Contrasting with the trends for open surgery, as illustrated in Figure 4, urgent admission rates for endovascular revascularizations for the whole of Brazil exhibited a non-static (p < 0.05) and rising (b > 0) trend over the period from 2010 to 2020. Figure 5 illustrates the admission rates by region.

The in-hospital mortality related to endovascular procedures over the period analyzed was 1.56%. The North region had the highest in-hospital mortality rate (3.52%), while the South region had the lowest rate (1.41%) for the same period.

**Figure 6:**
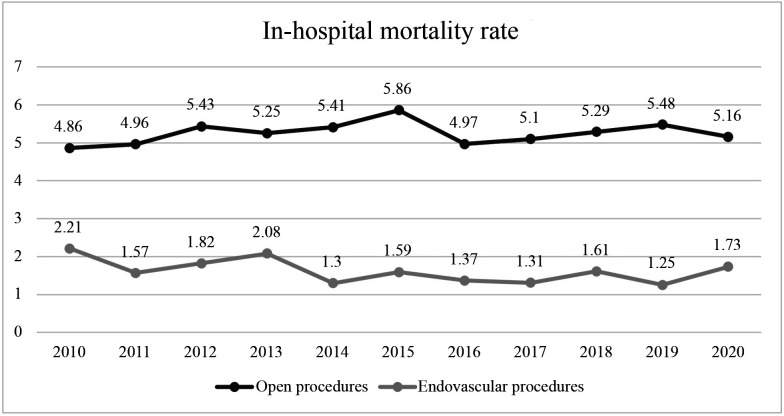
In-hospital mortality rates for urgent open and endovascular revascularizations in Brazil from 2010 to 2020.

Figure 6 shows a comparison between the in-hospital mortality rates for conventional and endovascular surgical procedures conducted in Brazil. Both rates had a static trend over time (p > 0.05), but the in-hospital mortality rate for open procedures was higher than the rate for endovascular procedures.

**Figure 7:**
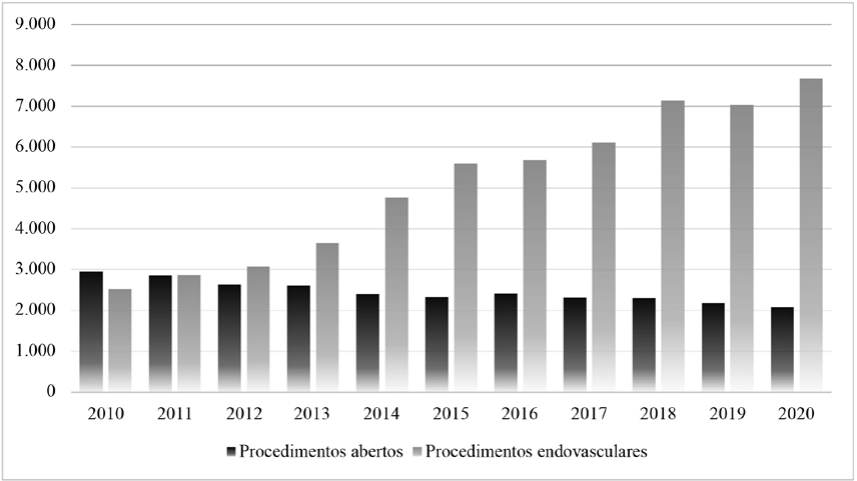
Distribution of numbers of urgent open and endovascular revascularizations in Brazil from 2010 to 2020.

Additionally, Figure 7 illustrates the relationship between the numbers of open and endovascular revascularizations conducted annually in Brazil over the study period.

**Figure 8:**
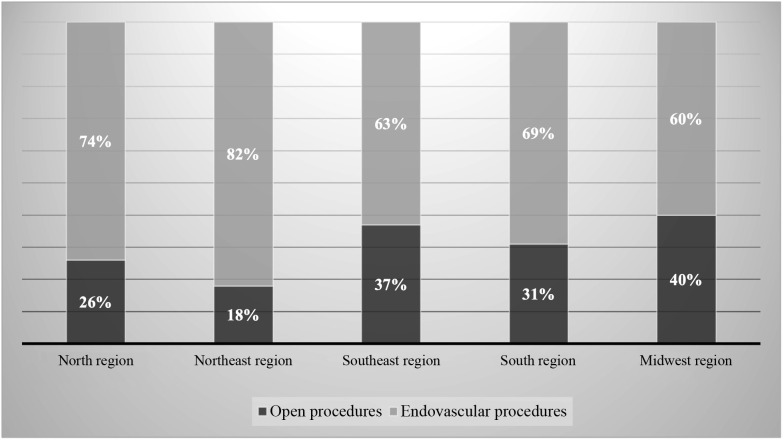
Percentages (%) of open and endovascular procedures by region from 2010 to 2020.

Finally, Figure 8 illustrates the relationships between the percentages of procedures that were open or endovascular in each region of Brazil, showing that the Northeast region had the highest proportion of endovascular surgery (82%) when compared to conventional procedures (18%), followed by the North region (74% vs. 26%), South region (69% vs. 31%), Southeast region (63% vs. 37%) and, finally, the Midwest region (60% vs. 40%).

## DISCUSSION

Critical ischemia is associated with shorter life expectancy, a significant reduction in the ability to walk, and a high probability of limb loss. Surgical approaches to treatment comprise endovascular surgery, open surgery, combined or hybrid procedures, and major amputations. Of these, endovascular techniques have been widely adopted because of their safety, effectiveness, and reliability.^8^^,^^10^

This study evaluated the numbers of open and endovascular revascularizations performed in Brazil from 2010 to 2020, showing that there was a greater number of admissions for percutaneous techniques, with around twice as many endovascular surgeries than open surgeries (56,132 vs. 27,086, respectively).

The temporal analysis of rates of admission for open procedures revealed a falling trend, whereas the trend for endovascular procedures was rising, for all regions of Brazil over the period analyzed.

Similarly, studies show that the number of endovascular procedures critical ischemia increased threefold from 1996 to 2006, while there was a 42% reduction in the number of open procedures. Other data show that the percentage of endovascular treatments rose from 13.4% to 27.4% from 2001 to 2011.^9^

Moreover, the data from this study show an unequal distribution across the five regions of Brazil. The South region had the highest admission rates in Brazil and the North region had the lowest rates of admission for surgical and endovascular interventions.

Analysis of the percentages of open and endovascular procedures showed a higher proportion of endovascular surgeries than open procedures in all regions of Brazil, with the highest proportion in the Northeast region (82% vs. 18%), while the predominance of endovascular over open revascularization was least pronounced in the Midwest region (60% vs. 40%).

In line with this, a study evaluating patients admitted for critical ischemia from 2003 to 2011 showed a significant reduction in the proportion of patients who underwent surgical revascularization (13.9% in 2003 to 8.8% in 2011), accompanied by a corresponding increase in endovascular revascularization over the same period (5.1% in 2003 to 11.0% in 2011). Another study conducted in Brazil showed a significant change in the proportions of treatment modalities used for peripheral arterial disease over the years 2008, 2010, and 2012, with a 57% increase in endovascular procedures and a 9.8% reduction in clinical treatment, whereas conventional surgical treatment remained stable.^11^^,^^12^

Management of peripheral arterial disease is costly for the healthcare system. This study showed that R$ 333,989,523.17 was spent in Brazil on admissions for open and endovascular revascularizations over the period analyzed, which equates to 0.75% of the total amount spent on admissions for urgent surgical procedures during the period.

Nascimento et al.^12^ demonstrated that the total cost of PAOD treatment on the public healthcare system increased by 37% from 2008 to 2012, with a sharp increase in costs related to endovascular procedures (92%) compared to the increases in the costs of conventional surgery (11%) and clinical treatment (30%).

Comparing the two procedures, the total cost of endovascular admissions was around 2.5 times greater than the cost of open revascularization in the present study. This is primarily because of the increase in the number of endovascular procedures over the period analyzed (approximately double the number of admissions recorded). However, it is also important to consider the higher cost of the materials employed for percutaneous techniques, which very often have to be imported, in addition to the fact that the database used for this study does not enable analysis of reinterventions.

Some studies have shown lower patency rates and greater likelihood of reintervention in patients treated with endovascular procedures, which could indirectly counterbalance the proportional costs of endovascular and open procedures over time.^12^^,^^13^

Additionally, it is important to point out that the total expenditure recorded on the database should be considered as the approved value and does not necessarily correspond to the sums actually paid out to the healthcare providers, since, depending on the situation, these units may receive budgetary resources, or there may be sums withheld or incentives paid that are not included in the data used for this study.

Moreover, the mean length of hospital stay was 10.2 days for admissions for open revascularization, whereas the mean stay for endovascular procedures was 5.3 days.

A multicenter study was conducted from 2013 to 2016 to assess the different strategies for treatment of patients admitted with PAOD, showing that the total length of hospital stay was significantly shorter for endovascular procedures when compared with open surgery (3.4 vs. 10 days, respectively). Moreover, it found that the adjusted cost of an endovascular procedure plus hospital stay was 42.3% less than the cost of open revascularization and was 57.3% less than the costs involved in a major amputation, because of the shorter length of hospital stay and reduced use of intensive care services related to endovascular procedures.^14^

Analyzing hospital mortality related to these procedures, the present study observed a rate of 5.24% for admissions for open revascularization, whereas the in-hospital mortality rate for endovascular procedures in Brazil was only 1.56% during the period analyzed.

Tang et al.^10^ conducted a meta-analysis comparing the two types of procedures in patients with PAOD and demonstrated a significantly higher overall mortality rate for open procedures when compared to endovascular treatment (10.86% vs. 7.54%, respectively), in addition to a shorter length of hospital stay, lower rate of complications (9.48% vs. 13.60%), and lower amputation rate (12.49% vs. 18.28%) among patients treated with endovascular surgery when compared to those who underwent conventional surgery. However, there were no statistically significant differences in survival rates or limb salvage at 30-day, 1-year, or 3-year follow-up.

Compared to open surgery, Agarwal et al.^11^ showed that the endovascular technique was associated with a significant reduction in intrahospital mortality (2.34% vs. 2.73%, p < 0.001), a reduced length of hospital stay (8.7 vs. 10.7 days, p < 0.001), and lower hospital costs ($31.679 vs. $32.485, p < 0.001), although both techniques had similar major amputation rates (6.5% vs. 5.7%, p = 0.75).

Excellent limb salvage rates and low perioperative morbidity and mortality have been reported as use of endovascular treatment has become widespread. However, questions have been raised about its durability, costs, and applications.^15^

While endovascular procedures are considered less expensive over the short term, the long-term comparison of percutaneous procedures and open revascularization in terms of costs and patient-centered results remain uncertain.^16^

To date, the BASIL study is the only completed prospective, randomized, and controlled study that has compared endovascular techniques with surgical revascularization in patients with critical ischemia. This trial assessed 452 patients with 3-year follow-up and did not find a difference between the two groups in the primary outcome of amputation-free survival (57% for open surgery vs. 52% for endovascular treatment). There was also no significant difference in relation to cost or long-term quality of life. However, the study had several limitations, since it only analyzed intraluminal angioplasty, excluding other technologies, such as stenting, and did not describe the influence of the pattern of arterial lesions.^15^^,^^17^^,^^18^

Modern tools for endovascular treatment are more sophisticated than in the past and there is no doubt that costs have increased with the new generation of guidewires, balloons, drug-eluting stents, and other more modern materials. While the new technology has the potential to improve technical success and durability, there is a financial cost that must be taken into consideration.^14^

The persistent clinical equilibrium, in combination with the scarcity of data on comparative efficacy to guide treatment of critical ischemia, has stimulated a multidisciplinary effort to organize a new prospective, randomized, and controlled multicenter trial designed to compare the efficacy of treatment, results, quality of life, and costs in patients with critical limb ischemia treated with open or endovascular revascularization (the Best Endovascular vs. Best Surgical Therapy – BEST-CLI trial). This trial is still ongoing and it is hoped that its results can guide surgeons in management of patients with critical ischemia.^9^^,^^15^

Finally, the data presented in this study were extracted from a national database that only includes procedures performed on the public healthcare system and are subject to the limitations of the errors and imprecisions inherent to a public registry and do not include data from Brazil’s private healthcare sector. In view of this, the results should not be extrapolated to the entire Brazilian population, due to the socioeconomic and cultural differences between populations dependent on public and private healthcare.

As such, the results are related to the subset of the Brazilian population that is dependent on the SUS and it is impossible to define the exact percentage of the Brazilian sample assessed in the study. It is also possible that the different regions of the country have different proportions of their populations dependent on the public healthcare system, which could impact on the analysis of the data.

Moreover, the number of reinterventions, i.e., the number of repeat procedures in the same patient, could not be filtered in the records. Other limitations of the study include the lack of availability of data on the limb salvage rates of each technique and the inability to differentiate between procedures conducted after trauma or acute arterial emboli and those due to PAOD.

## CONCLUSIONS

This study showed that there was a predominance of endovascular urgent revascularizations on the public healthcare system from 2010 to 2020 in all regions of Brazil.

Over the period analyzed, there was a falling trend for open procedures and a rising trend for endovascular interventions. Additionally, admissions for endovascular revascularizations had a shorter length of hospital stay and a smaller in-hospital mortality rate than admissions for open revascularization.
