# Supplementary material for: Peripheral artery disease: a comparison of urgent open and endovascular revascularizations on the public health system in Brazil, from 2010 to 2020
Source: J Vasc Bras. 2022 Jul 29;21:e20220016. doi: 10.1590/1677-5449.202200161 (PMC9351983; doi:10.1590/1677-5449.202200161)
Supplement: Translation

# Translation: Doença arterial obstrutiva periférica: um estudo comparativo entre revascularizações abertas e endovasculares realizadas em caráter de urgência no sistema público de saúde do Brasil entre 2010 e 2020

**DOI:** 10.1590/1677-5449.202200162

**Authors:** Thaís Rodrigues Magalhães, Daniel César Magalhães Fernandes, Roberto Gomide, Hideki Nakano, André Vespasiano Afiune, Rômulo Mendes Silva, Paulo Ricardo Alves Moreira, Rosa Tanmirys de Sousa Lima

**Affiliations:** 1 Hospital Geral de Goiânia Dr. Alberto Rassi – HGG, Goiânia, GO, Brasil.

**Keywords:** doença arterial obstrutiva periférica, revascularização aberta, cirurgia endovascular

## Abstract

**Contexto:**

A doença arterial obstrutiva periférica apresenta alta prevalência, sendo associada a elevado risco de eventos cardiovasculares. A intervenção cirúrgica ou endovascular faz-se necessária na isquemia crítica do membro.

**Objetivos:**

Avaliar a distribuição de realização de revascularizações abertas e endovasculares nas diferentes regiões do Brasil, analisando os custos para o sistema de saúde e a mortalidade relacionada a esses procedimentos.

**Métodos:**

Foi realizado um estudo epidemiológico observacional transversal descritivo para avaliar as cirurgias abertas e endovasculares realizadas no sistema público de saúde do Brasil entre 2010 e 2020. Os dados foram coletados através do Departamento de Informática do SUS (Datasus).

**Resultados:**

No período analisado, foram registradas 83.218 internações para realização de cirurgias abertas e endovasculares, com um custo total de R$ 333.989.523,17. Houve predominância das internações para os procedimentos percutâneos (56.132) em relação aos cirúrgicos convencionais (27.086). As Regiões Sudeste e Sul concentraram a maior parte do total de procedimentos realizados no país (83%), enquanto a Região Norte foi a que apresentou a menor taxa de internação. Observou-se uma tendência decrescente para os procedimentos abertos, e uma tendência crescente para os endovasculares. A média de permanência hospitalar foi menor nos procedimentos endovasculares (5,3 dias) em relação aos abertos (10,2 dias). Além disso, notou-se uma maior taxa de mortalidade hospitalar relacionada à revascularização aberta em relação à endovascular (5,24% *versus* 1,56%).

**Conclusões:**

As técnicas endovasculares consistiram em uma abordagem dominante no tratamento cirúrgico da isquemia crítica, apresentando menor taxa de mortalidade hospitalar e menor tempo de internação quando comparada às cirurgias abertas.

## INTRODUÇÃO

A doença arterial obstrutiva periférica (DAOP) é decorrente de fenômenos ateroscleróticos sistêmicos, que provocam obstruções arteriais, está associada a alta morbimortalidade cardiovascular e acomete uma grande parte da população, gerando considerável sofrimento humano e prejuízo econômico^1^^,^^2^.

Entre 2000 e 2010, a prevalência de DAOP aumentou em 13,1% em países de alta renda, e em 28,7% nos países de média e baixa renda^3^^,^^4^.

O tratamento cirúrgico estaria indicado para pacientes com isquemia crítica do membro, seja por dor em repouso ou presença de lesão trófica, e para pacientes com claudicação intermitente que não responderam ao tratamento clínico, apresentando limitações na qualidade de vida e/ou na vida profissional, além de condições anatômicas favoráveis ao procedimento^1^.

Os pacientes candidatos ao tratamento cirúrgico podem ser submetidos a revascularização aberta ou endovascular e, caso não haja possibilidade de salvamento do membro, a amputações maiores primárias ou secundárias^5^^-^8.

Historicamente, a revascularização era considerada o padrão-ouro para os pacientes com doença arterial oclusiva periférica, mostrando excelentes taxas de salvamento de membro e durabilidade^9^.

Entretanto, nas últimas duas décadas, uma revolução endovascular levou a um aumento significativo na utilização das técnicas percutâneas para revascularização do membro isquêmico, com inúmeros relatos de excelentes taxas de salvamento do membro. Além disso, quando comparada às técnicas abertas, a terapia endovascular apresentou uma menor morbidade e mortalidade perioperatória^9^.

Dessa forma, torna-se importante conhecer a distribuição das cirurgias abertas e endovasculares, levantando questões quanto às taxas de internação, custos para o sistema de saúde e mortalidade relativa a esses procedimentos. Nesse sentido, o objetivo do estudo consistiu em realizar uma análise comparativa das revascularizações cirúrgicas e endovasculares realizadas pelo sistema público de saúde nas diferentes regiões do Brasil entre 2010 e 2020.

## MÉTODO

A pesquisa caracterizou-se como um estudo epidemiológico observacional transversal descritivo, cujos dados foram coletados a partir de informações disponibilizadas pelo banco de dados do Ministério da Saúde, o Datasus (Departamento de Informática do SUS). Através da seção *TABNET*, foi selecionada a opção “Assistência à Saúde” e, posteriormente, “Produção Hospitalar (SIH/SUS)”, sendo escolhidos os “Dados consolidados AIH (RD), por local de internação a partir de 2008”, e selecionadas, na abrangência geográfica, as diferentes regiões e unidades da federação do Brasil.

Foram coletadas informações das internações relacionadas a revascularizações abertas e endovasculares no período de janeiro de 2010 a dezembro de 2020, sendo selecionados os pacientes internados em caráter de urgência, de forma a avaliar os procedimentos realizados nos pacientes com provável isquemia crítica do membro.

Os códigos de procedimentos selecionados relativos às revascularizações abertas foram: 0406020310; 0406020329; 0406020345; 0406020353; 0406020361; 0406020388; 0406020450; 0406020442; 0406020434. Já os códigos relacionados aos procedimentos endovasculares consistiram nos seguintes: 0406040281; 0406040028; 0406040044; 0406040052; 0406040060; e 0406040079. Os códigos de amputações não foram incluídos devido à impossibilidade de diferenciar, na base de dados de acesso livre do Datasus, os procedimentos traumáticos daqueles provocados por isquemia crítica.

Foram também coletados os dados a respeito da média de permanência hospitalar, valor total gasto com as internações e taxa de mortalidade relacionada aos procedimentos realizados nesse mesmo período. Além disso, foi calculada a taxa de internação através da divisão do número de internações pela população residente relativa ao ano e região, disponibilizada pelo Instituto Brasileiro de Geografia e Estatística (IBGE), sendo avaliada a cada 100.000 habitantes. Os resultados encontrados foram organizados na forma de tabelas e gráficos.

Para a análise temporal dos resultados, foi utilizado o método de Prais-Winsten, um modelo de regressão linear executado através do software Stata, de forma a avaliar a tendência dos resultados como crescente, decrescente ou estacionária, através do cálculo do valor de p (maior ou menor que 0,05) e valor de b (maior ou menor que 0).

A aprovação pelo Comitê de Ética em Pesquisa foi dispensada, uma vez que os dados obtidos são de domínio público, acesso livre e irrestrito, sem identificação individual dos pacientes.

## RESULTADOS

No Brasil, durante o período de 2010 a 2020, os dados mostraram um total de 83.218 internações para realização de revascularizações abertas e endovasculares em caráter de urgência, com um valor total gasto de R$ 333.989.523,17, relativo às internações e aos procedimentos realizados nesse mesmo período. Conforme mostrado na Tabela 1, o número de internações e os custos hospitalares foram maiores na Região Sudeste, e menores na Região Norte do Brasil.

**Tabela 1:** Relação do número de internações e o valor total das revascularizações abertas e endovasculares realizadas em caráter de urgência por regiões do Brasil durante o período de 2010 a 2020.

**Região**	**Internações**	**Valor total (em reais)**
Região Norte	881	R$ 3.161.867,43
Região Nordeste	9.542	R$ 34.663.032,26
Região Sudeste	36.548	R$ 152.446.124,24
Região Sul	32.314	R$ 127.179.973,15
Região Centro-Oeste	3.933	R$ 16.538.526,09
**T**otal	83.218	R$ 333.989.523,17

Fonte: Ministério da Saúde. Sistemas de Informações Hospitalares do SUS (SIH/SUS).

A Figura 1 representa a frequência relativa aproximada do total de revascularizações abertas e endovasculares realizadas em cada região brasileira. Percebe-se que a maior parte (83%) das internações no país estiveram concentradas nas Regiões Sudeste e Sul, e que a Região Norte representou apenas com 1% do total de procedimentos.

**Figura 1:** Frequência relativa do total dos procedimentos abertos e endovasculares realizados por regiões do Brasil, no período de 2010 a 2020.

Em relação às revascularizações abertas, a Tabela 2 mostra que foram aprovadas 27.086 internações para a realização de cirurgias abertas no Brasil entre 2010 e 2020, com um custo total de R$ 94.614.711,94.

**Tabela 2:** Número de internações, tempo médio de permanência hospitalar, taxa de mortalidade e valor total gasto em relação às revascularizações abertas realizadas em caráter de urgência por regiões do Brasil, durante o período de 2010 a 2020.

**Região**	**Internações**	**Média de permanência (dias)**	**Taxa de mortalidade (%)**	**Valor total** **(reais)**
Região Norte	228	13,3	7,89	R$ 674.170,87
Região Nordeste	1.755	13,1	5,87	R$ 6.355.481,17
Região Sudeste	13.420	9,5	5,98	R$ 49.605.675,58
Região Sul	10.119	9,4	4,08	R$ 32.504.387,60
Região Centro-Oeste	1.564	17,1	5,31	R$ 5.474.996,72
**Total**	27.086	10,2	5,24	R$ 94.614.711,94

Fonte: Ministério da Saúde. Sistemas de Informações Hospitalares do SUS (SIH/SUS).

Novamente, as Regiões Sudeste e Sul foram as que apresentaram a maior quantidade de revascularizações abertas, representando, de forma conjunta, 87% do total das cirurgias realizadas no Brasil. A Região Norte ficou em último lugar, realizando apenas 228 procedimentos abertos no período estipulado. Da mesma forma, o valor total gasto foi condizente com o número de internações e de procedimentos, sendo maior na Região Sudeste e menor na Região Norte.

Em relação à média de permanência hospitalar nas internações por revascularizações abertas, a Região Centro-Oeste foi a que apresentou o maior tempo de internação (17,1 dias), e a Região Sul apresentou a menor média (9,4 dias). No Brasil, o tempo médio de permanência hospitalar nas cirurgias abertas foi de 10,2 dias.

Analisando a mortalidade relativa aos procedimentos abertos, obteve-se uma taxa de mortalidade hospitalar de 5,24% no Brasil durante o período de 2010 a 2020. Apesar do menor número de cirurgias realizadas, a Região Norte foi a que apresentou a maior taxa de mortalidade (7,89%), seguida pela Região Sudeste (5,98%), Região Nordeste (5,87%), Região Centro-Oeste (5,31%) e Região Sul (4,08%).

Por último, ao fazer o cálculo da taxa de internação para realização dos procedimentos abertos em caráter de urgência, conforme mostrado na Figura 2, observou-se uma tendência não estacionária (p < 0,05) e decrescente (b < 0) no Brasil, durante o período analisado. Já a Figura 3 mostra as taxas de internação em caráter de urgência a cada ano distribuídas nas diferentes regiões do país.

**Figura 2:** Taxas de internação para revascularizações abertas realizadas em caráter de urgência no Brasil durante o período de 2010 a 2020.

**Figura 3:** Distribuição das taxas de internação para revascularizações abertas realizadas em caráter de urgência nas diferentes regiões do Brasil, durante o período de 2010 a 2020.

Em relação aos procedimentos endovasculares, conforme a Tabela 3, foram realizadas, entre 2010 e 2020, 56.132 internações no território brasileiro, com um custo total de R$ 239.374.811,23.

**Tabela 3:** Número de internações, tempo médio de permanência hospitalar, taxa de mortalidade e valor total gasto em relação às cirurgias endovasculares realizadas em caráter de urgência por regiões do país, durante o período de 2010 a 2020.

**Região**	**Internações**	**Média de permanência (dias)**	**Taxa de mortalidade (%)**	**Valor total (reais)**
Região Norte	653	8,9	3,52	R$ 2.487.696,56
Região Nordeste	7.787	7,5	1,81	R$ 28.307.551,09
Região Sudeste	23.128	4,2	1,47	R$ 102.840.448,66
Região Sul	22.195	5,1	1,41	R$ 94.675.585,55
Região Centro-Oeste	2.369	9,1	2,57	R$ 11.063.529,37
**Total**	56.132	5,3	1,56	R$ 239.374.811,23

Fonte: Ministério da Saúde. Sistemas de Informações Hospitalares do SUS (SIH/SUS).

As Regiões Sudeste e Sul foram responsáveis pela maior parte dos procedimentos do país (80,7%), enquanto a Região Norte concentrou apenas 1,2% das angioplastias realizadas.

Nas internações para os procedimentos endovasculares, a média de permanência hospitalar no Brasil foi de 5,3 dias, sendo maior na Região Centro-Oeste (9,1 dias), e menor na Região Sudeste (4,2 dias).

Ao contrário das cirurgias abertas, o cálculo da taxa de internação para as revascularizações endovasculares em caráter de urgência, conforme demonstrado na Figura 4, mostrou uma tendência não estacionária (p < 0,05) e crescente (b > 0) no Brasil, durante o período de 2010 a 2020. A Figura 5 mostra as respectivas taxas de internação distribuídas nas diferentes regiões do país.

**Figura 4:** Taxas de internação para revascularizações endovasculares realizadas em caráter de urgência no Brasil, durante o período de 2010 a 2020.

**Figura 5:** Distribuição das taxas de internação para revascularizações endovasculares realizadas em caráter de urgência nas diferentes regiões do Brasil, durante o período de 2010 a 2020.

A taxa de mortalidade hospitalar relativa aos procedimentos endovasculares no período estipulado foi de 1,56%. A Região Norte apresentou maior taxa de mortalidade hospitalar (3,52%), enquanto a Região Sul mostrou a menor taxa (1,41%) nesse mesmo período.

A Figura 6 mostra um comparativo entre as taxas de mortalidade hospitalar dos procedimentos cirúrgicos convencionais e endovasculares realizados no Brasil. Ambas as taxas apresentaram tendência estacionária (p > 0,05) no decorrer do tempo; porém, a taxa de mortalidade hospitalar dos procedimentos abertos foi superior à taxa dos procedimentos endovasculares.

**Figura 6:** Taxas de mortalidade das revascularizações abertas e endovasculares realizadas em caráter de urgência no Brasil, durante o período de 2010 a 2020.

Adicionalmente, a Figura 7 mostra a relação anual do número de revascularizações abertas e endovasculares no Brasil durante o período estipulado.

**Figura 7:** Distribuição da quantidade de Revascularizações abertas e endovasculares realizadas em caráter de atendimento de urgência no Brasil, durante o período de 2010 a 2020.

Finalmente, a Figura 8 traz a relação percentual da quantidade de procedimentos abertos e endovasculares realizados por cada região brasileira, mostrando que a Região Nordeste foi a que apresentou maior proporção de cirurgias realizadas pela técnica endovascular (82%) quando comparada às técnicas convencionais (18%), seguida pela Região Norte (74% *versus* 26%), Região Sul (69% *versus* 31%), Região Sudeste (63% *versus* 37%) e, por último, a Região Centro-Oeste (60% *versus* 40%).

**Figura 8:** Relação em percentual (%) dos procedimentos abertos e endovasculares em cada região brasileira, durante o período de 2010 a 2020.

## DISCUSSÃO

A isquemia crítica está associada a menor expectativa de vida, redução significativa na deambulação e alta probabilidade de perda do membro. As abordagens cirúrgicas para o seu tratamento consistem em cirurgia endovascular, cirurgia aberta, procedimentos combinados ou híbridos e amputações maiores. Entre elas, a técnica endovascular tem sido largamente utilizada, devido à sua segurança, eficiência e confiabilidade^8^^,^^10^.

Ao avaliar a quantidade de revascularizações abertas e endovasculares realizadas no Brasil entre 2010 e 2020, o presente estudo mostrou uma superioridade no número de internações para realização da técnica percutânea, com um valor cerca de duas vezes maior das cirurgias endovasculares em relação às cirurgias abertas (56.132 *versus* 27.086, respectivamente).

Na análise temporal da taxa de internação para os procedimentos abertos, observou-se uma tendência decrescente, enquanto, nos procedimentos endovasculares, essa tendência foi crescente em todo o território brasileiro, durante o período analisado.

De forma semelhante, alguns estudos mostraram que, de 1996 a 2006, houve um aumento de três vezes no número de procedimentos endovasculares para isquemia crítica, com uma redução de 42% no número de procedimentos abertos. Outros dados mostraram que, de 2001 a 2011, houve um aumento no percentual de tratamentos endovasculares de 13,4% para 27,4%^9^.

Além disso, os dados deste estudo mostraram uma distribuição desigual entre as diferentes regiões do país. A Região Sul foi a que apresentou as maiores taxas de internação, e a Região Norte concentrou as menores taxas de internação para realização de intervenções cirúrgicas e endovasculares no território brasileiro.

Avaliando a relação percentual dos procedimentos abertos e endovasculares, os resultados mostraram maior proporção de cirurgias endovasculares em relação às abertas em todas as regiões brasileiras, sendo a Região Nordeste a que apresentou a maior proporção (82% *versus* 18%), enquanto a Região Centro-Oeste foi a que demonstrou um menor número de cirurgias endovasculares em relação às revascularizações abertas (60% *versus* 40%).

Consoante a isso, um estudo avaliando pacientes internados com isquemia crítica entre 2003 e 2011 mostrou uma redução significativa na proporção de pacientes submetidos a revascularização cirúrgica (13,9% em 2003 para 8,8% em 2011), acompanhada de um aumento correspondente de revascularização endovascular no mesmo período (5,1% em 2003 para 11,0% em 2011). Outro estudo realizado em território brasileiro mostrou uma mudança significativa na proporção das modalidades de tratamento para doença arterial periférica ao longo dos anos de 2008, 2010 e 2012, com um aumento de 57% dos procedimentos endovasculares e uma redução de 9,8% em relação ao tratamento clínico, enquanto a abordagem cirúrgica convencional permaneceu estável^11^^,^^12^.

O manejo da doença arterial periférica traz custos ao sistema de saúde. O presente estudo mostrou que, no período analisado, foram gastos R$ 333.989.523,17 no Brasil com internações para realização de revascularizações abertas e endovasculares, o equivalente a 0,75% do valor total gasto com internações para procedimentos cirúrgicos em caráter de urgência no mesmo período.

Nascimento et al.^12^ demonstraram que o custo total do tratamento de DAOP para o sistema público de saúde aumentou em 37% de 2008 a 2012, com acentuado aumento dos custos relativos aos procedimentos endovasculares (92%), quando comparado ao incremento nos custos das cirurgias convencionais (11%) e do tratamento clínico (30%).

Comparando os dois procedimentos, no presente estudo, o custo total das internações endovasculares foi cerca de 2,5 vezes maior em relação às revascularizações abertas. Esse dado está principalmente relacionado ao incremento no número de procedimentos endovasculares durante o período analisado (aproximadamente o dobro de internações registradas). Porém, é importante levar em consideração o maior custo do material utilizado na técnica percutânea, muitas vezes necessitando de importação, além da impossibilidade de avaliação das reintervenções no banco de dados disponível para este estudo.

Alguns estudos mostram menor patência e maior possibilidade de reintervenções nos pacientes submetidos aos procedimentos endovasculares, o que pode contrabalancear indiretamente os custos proporcionais dos procedimentos endovasculares e abertos ao longo do tempo^12^^,^^13^.

Além disso, é importante destacar que o valor total gasto relatado pela base de dados deve ser considerado como o valor aprovado da produção e não obrigatoriamente corresponde ao valor repassado aos estabelecimentos de saúde, uma vez que, dependendo da situação, as unidades recebem recursos orçamentários, ou pode haver retenções e pagamentos de incentivos, não apresentados nos dados do estudo.

Ademais, nas internações para realização de revascularizações abertas, a média de permanência hospitalar foi de 10,2 dias, enquanto o tempo médio de internação nos procedimentos endovasculares foi de 5,3 dias.

Foi realizado um estudo multicêntrico entre os anos de 2013 e 2016 para avaliar as diferentes estratégias de tratamento dos pacientes admitidos com DAOP, o qual mostrou que o tempo de internação total foi significativamente menor para procedimentos endovasculares quando comparados a cirurgias abertas (3,4 *versus* 10 dias, respectivamente). Além disso, encontrou-se que o custo ajustado de realizar um procedimento endovascular e da permanência hospitalar foi 42,3% menor que o custo de revascularização aberta e 57,3% menor que o custo associado à realização de amputação maior, devido ao menor tempo de internação e à menor utilização de serviços das unidades de terapia intensiva observados nos procedimentos endovasculares^14^.

Analisando a mortalidade hospitalar relativa aos procedimentos, observou-se, no presente estudo, uma taxa de 5,24% nas internações para revascularizações abertas, enquanto a taxa de mortalidade hospitalar dos procedimentos endovasculares foi de apenas 1,56% no Brasil, durante o período analisado.

Tang et al.^10^ realizaram uma metanálise comparando os dois tipos de procedimentos nos pacientes com DAOP e demonstraram uma taxa de mortalidade geral significativamente maior nos procedimentos abertos quando comparados aos endovasculares (10,86% *versus* 7,54%, respectivamente), além de menor tempo de internação hospitalar, menores taxas de complicações (9,48% *versus* 13,60%) e menor taxa de amputação (12,49% *versus* 18,28%) nos pacientes submetidos a cirurgia endovascular quando comparados aos submetidos a técnica convencional. Entretanto, não houve diferença estatisticamente significativa em relação às taxas de sobrevida ou de salvamento do membro após 30 dias, 1 ano e 3 anos de acompanhamento.

Comparada à cirurgia aberta, Agarwal et. al. mostraram que a técnica endovascular foi associada a uma redução significativa de mortalidade intra-hospitalar (2,34% *versus* 2,73%, p < 0,001), além de diminuição do tempo de internação (8,7 *versus* 10,7 dias, p < 0,001) e dos custos da hospitalização ($31,679 *versus* $32,485, p < 0,001), apesar de ambas técnicas apresentarem taxas de amputação maior semelhantes (6,5% *versus* 5,7%, p = 0,75)^11^.

Excelentes taxas de salvamento do membro e baixa morbimortalidade perioperatória têm sido reportadas com a expansão do tratamento endovascular. Entretanto, questões quanto à durabilidade, custos e aplicação também têm sido levantadas^15^.

Embora os procedimentos endovasculares sejam considerados menos caros a curto prazo, a comparação a longo prazo entre os procedimentos percutâneos e as revascularizações abertas quanto aos custos e resultados centrados no paciente ainda permanece incerta^16^.

Até o momento, o estudo BASIL é o único estudo prospectivo, randomizado e controlado já completo que comparou as técnicas endovasculares com revascularização cirúrgica em pacientes com isquemia crítica. Esse ensaio avaliou 452 pacientes com um seguimento de 3 anos, não encontrando diferença no desfecho primário de sobrevida livre de amputação entre os dois grupos (57% na cirurgia aberta *versus* 52% endovascular). Também não foi encontrada diferença significativa em relação aos custos e à qualidade de vida a longo prazo. Entretanto, esse estudo apresentou várias limitações, uma vez que avaliou apenas as terapias de angioplastia intraluminal, excluindo outras tecnologias. como colocação de stents, além de não descrever a influência do padrão das lesões arteriais^15^^,^^17^^,^^18^.

As ferramentas modernas da terapia endovascular são mais sofisticadas do que as do passado, e não há dúvida de que os custos têm aumentado com a nova geração de fio-guias, balões, stents farmacológicos e outros materiais mais modernos. Embora a nova tecnologia tenha o potencial de melhorar o sucesso técnico e a durabilidade, existe um custo financeiro que deve ser levado em consideração^14^.

O persistente equilíbrio clínico, em combinação com a escassez de dados de eficácia comparativa para guiar o tratamento da isquemia crítica, tem levado a um esforço multidisciplinar para organizar um novo ensaio prospectivo, randomizado, controlado e multicêntrico, desenhado para comparar a eficácia do tratamento, resultados, qualidade de vida e custos dos pacientes com isquemia crítica do membro submetidos a revascularização aberta ou endovascular (*Best Endovascular versus Best Surgical Therapy* – BEST-CLI *trial*). O ensaio clínico atualmente está em desenvolvimento, e espera-se que seus resultados guiem os cirurgiões no manejo dos pacientes com isquemia crítica^9^^,^^15^.

Por fim, os dados apresentados no presente estudo foram extraídos de um banco de dados nacional que inclui apenas os procedimentos realizados no sistema público de saúde, apresentando como limitações os erros e as imprecisões inerentes a um registro público e a não inclusão dos dados da saúde suplementar do Brasil. Sendo assim, os resultados não devem ser extrapolados para toda a população brasileira, devido às diferenças socioeconômicas e culturais entre as populações dependentes de saúde pública e suplementar.

Assim, os resultados obtidos estão relacionados à população brasileira dependente do SUS, não sendo possível definir a porcentagem exata da amostra brasileira avaliada no estudo, e com a possibilidade de que as diferentes regiões do país apresentem números distintos de população dependente de saúde pública, o que pode interferir na análise dos dados.

Além disso, o número de reintervenções, ou seja, procedimentos realizados de forma repetida em um mesmo paciente, não foi possível de ser filtrado no registro. Outras limitações do estudo consistiram na indisponibilidade de dados a respeito da taxa de salvamento do membro em cada técnica utilizada e da impossibilidade de diferenciar os procedimentos realizados após trauma ou embolia arterial aguda daqueles por DAOP.

## CONCLUSÃO

O presente estudo mostrou que, entre 2010 e 2020, houve uma predominância de revascularizações endovasculares no sistema público de saúde e em caráter de urgência em todas as regiões do Brasil.

No transcorrer do tempo analisado, observou-se uma tendência decrescente para os procedimentos abertos e uma tendência crescente para as intervenções endovasculares. Além disso, as internações para revascularizações endovasculares apresentaram menor tempo de internação hospitalar e menor taxa de mortalidade hospitalar quando comparadas às internações para revascularizações abertas.
